# Ultrasound Perfusion Imaging for the Detection of Cerebral Hypoperfusion After Aneurysmal Subarachnoid Hemorrhage

**DOI:** 10.1007/s12028-022-01460-z

**Published:** 2022-02-24

**Authors:** Christian Fung, Dieter Henrik Heiland, Raluca Reitmeir, Wolf-Dirk Niesen, Andreas Raabe, Jens Eyding, Oliver Schnell, Roland Rölz, Werner J. Z´Graggen, Jürgen Beck

**Affiliations:** 1 Department of Neurosurgery, Medical Center, University of Freiburg, Breisacher Strasse 64, 79106 Freiburg, Germany; 2 Medical Faculty, University of Freiburg, Freiburg, Germany; 3 Department of Neurosurgery, Inselspital, University of Bern, Bern, Switzerland; 4 Department of Neurology, Medical Center, University of Freiburg, Freiburg, Germany; 5 Department of Neurology, Gemeinschaftskrankenhaus Herdecke, University Witten/Herdecke, Herdecke, Germany; 6 Department of Neurology, Inselspital, University of Bern, Bern, Switzerland

**Keywords:** Subarachnoid hemorrhage, Perfusion, Ultrasonography, Vasospasm (intracranial)

## Abstract

**Background:**

Delayed cerebral ischemia increases mortality and morbidity after aneurysmal subarachnoid hemorrhage (aSAH). Various techniques are applied to detect cerebral vasospasm and hypoperfusion. Contrast-enhanced ultrasound perfusion imaging (UPI) is able to detect cerebral hypoperfusion in acute ischemic stroke. This prospective study aimed to evaluate the use of UPI to enable detection of cerebral hypoperfusion after aSAH.

**Methods:**

We prospectively enrolled patients with aSAH and performed UPI examinations every second day after aneurysm closure. Perfusion of the basal ganglia was outlined to normalize the perfusion records of the anterior and posterior middle cerebral artery territory. We applied various models to characterize longitudinal perfusion alterations in patients with delayed ischemic neurologic deficit (DIND) across the cohort and predict DIND by using a multilayer classification model.

**Results:**

Between August 2013 and December 2015, we included 30 patients into this prospective study. The left–right difference of time to peak (TTP) values showed a significant increase at day 10–12. Patients with DIND demonstrated a significant, 4.86 times increase of the left–right TTP ratio compared with a mean fold change in patients without DIND of 0.9 times (*p* = 0.032).

**Conclusions:**

UPI is feasible to enable detection of cerebral tissue hypoperfusion after aSAH, and the left–right difference of TTP values is the most indicative result of this finding.

## Introduction

Delayed cerebral ischemia (DCI) due to hypoperfusion in the course of cerebral vasospasm is, besides rebleeding, the leading cause of morbidity and mortality after initial aneurysmal rupture in subarachnoid hemorrhage (SAH) [1, 2]. Approximately 30% of patients with aneurysmal SAH (aSAH) develop DCI [3]. The pathogenesis of DCI is not fully attributable to large-vessel vasospasm alone, but rather hemodynamic alterations (e.g., impaired cerebral perfusion due to different mechanisms) is the common denominator [4, 5]. Because DCI is preceded by an impaired cerebral perfusion, using a noninvasive screening tool that measures cerebral perfusion to enable the detection of disturbances of the cerebral microcirculation rather than a surrogate parameter, such as blood flow velocities within the cerebral vessels, might be superior [6–8].

Recently, a proof-of-concept study showed the capability of cerebral ultrasound perfusion imaging (UPI) to determine normoperfused, hypoperfused, and nonperfused tissue in patients with acute ischemic stroke [9, 10]. UPI might be a useful screening tool in patients with SAH to assess cerebral perfusion. We performed this prospective study to evaluate the capability of contrast-enhanced UPI to enable the detection of cerebral hypoperfusion in patients with SAH.

## Methods

### Population and Study Design

We prospectively included patients with aSAH who were admitted to the Department of Neurosurgery at the University Hospital Bern between August 2013 and December 2015. Inclusion criteria were as follows: confirmed aSAH, admission within 72 h after SAH, 18 years of age or older, and written informed consent. Patients were excluded in cases of pregnancy, severe heart and lung disease, allergy to SonoVue, and severe renal insufficiency. The results of the perfusion measurement served as the primary end point. All patients were treated according to institutional protocols and international guidelines [11, 12], including aneurysm occlusion within 24 h after admission and administration of oral nimodipine. Initial UPI was performed (see UPI protocol as follows) after aneurysm occlusion and within 4 days after admission, and thereafter every second day during the 14-day period after ictus. A clinical assessment for the presence of a delayed ischemic neurologic deficit (DIND) was done prior to UPI measurement by the treating medical staff. The study was approved by the local ethics committee (Kantonale Ethikkommission Bern 174/12) and has been registered (identifier: NCT02907879). Informed consent was obtained from patients or next of kin.

### DIND

Delayed ischemic neurological deficit was defined as the occurrence of focal neurological impairment, such as hemiparesis, aphasia, apraxia, hemianopia, or neglect, or a decrease of at least 2 points on the Glasgow Coma Scale for at least 1 h that cannot be attributed to other causes by means of clinical assessment, computed tomography (CT), or magnetic resonance imaging of the brain and appropriate laboratory studies [13]. We used DIND as an outcome parameter rather than DCI because the rationale of UPI is to assess hypoperfusion to prevent cerebral ischemia.

### UPI

Ultrasound perfusion imaging was performed by a dedicated study team who were not masked for clinical course. The transcranial color duplex sonography was performed with a 1–5 MHz dynamic sector array (S5-1) from a Philips iU22 ultrasound machine (Philips Healthcare, Andover, MA). The field-of-view was set to an imaging depth of 150 mm in a sector angle of 90°. The imaging plane was then tilted to the diencephalic plane as described before, in which the frontal horns of the side ventricles and the third ventricle serve as landmarks and where the anterior and posterior middle cerebral artery (MCA) territory and the basal ganglia (BG) as region of interest could be identified without artifacts from major vessels [14]. High mechanical index bolus imaging was performed from both sides individually [14]. Data acquisition of 45 s was started immediately after intravenous (i.v.) injection of a 2-ml bolus of the second-generation contrast enhancer SonoVue (Bracco International, Milano, Italy), followed by a 10-ml flush of saline using an mechanical index (MI) setting of 1.34 and a frame rate of 5 Hz.

### Postprocessing Image Data of UPI

**Fig. 1:**
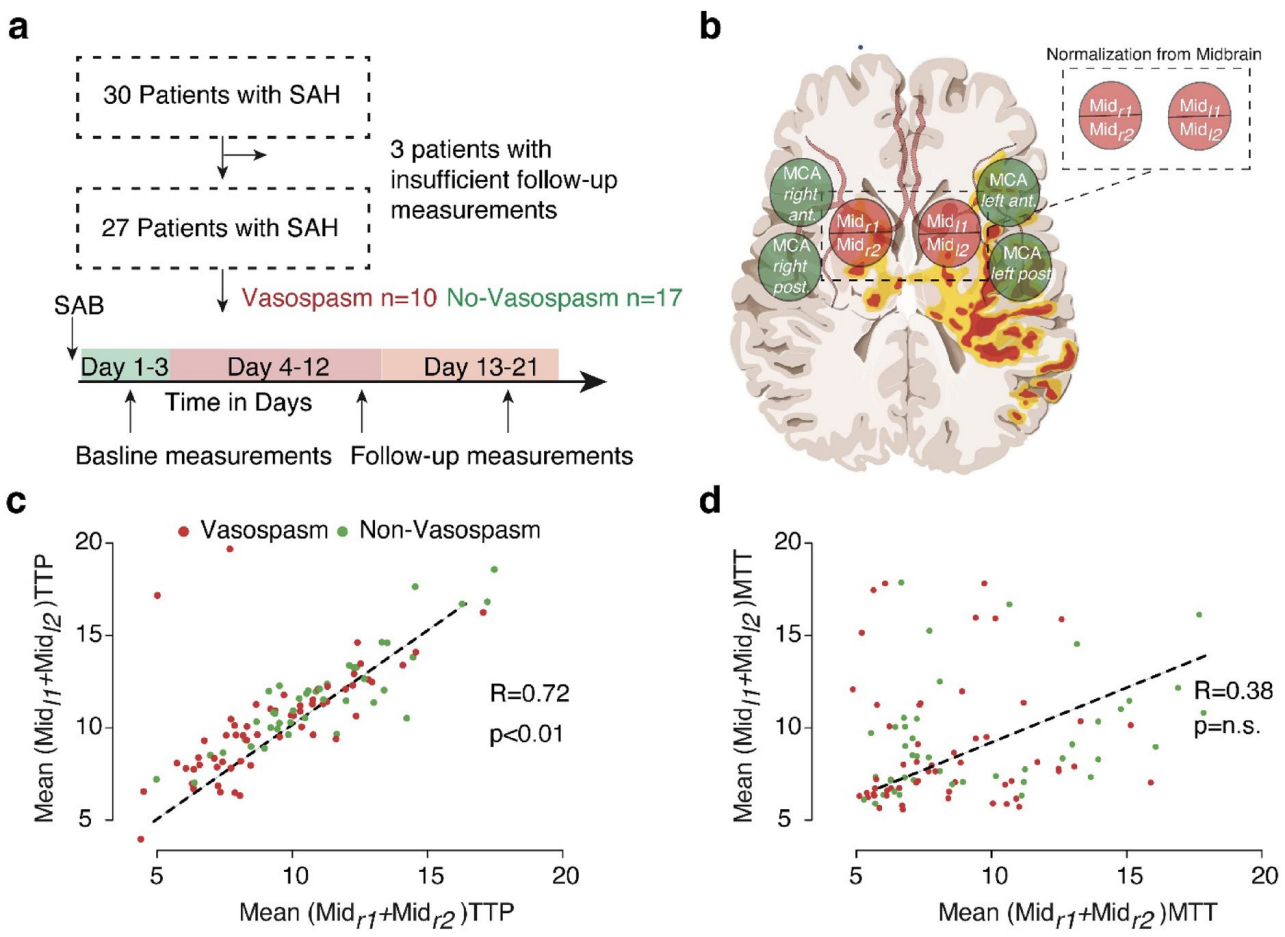
Illustration of the workflow of examined patients (**a**) and region of interests that were evaluated by postprocessing offline analysis (**b**). The graphs represent ultrasound perfusion measurements after normalization with the corresponding midline region for TTP (**c**) and MTT (**d**). MCA, middle cerebral artery, Mid, midline region, MTT, mean transit time, SAH, subarachnoid hemorrhage, TTP, time to peak

UPI data were analyzed offline by using VueBox 4.3 (Bracco Imaging, Geneva, Switzerland). A complete examination consisted of two single UPI examinations, one performed from the left side and one performed from the right side. Time to peak (TTP) and mean transit time (MTT) were quantified in four regions of interest (ROIs) per UPI examination: BG of the ipsilateral and contralateral hemisphere and anterior and posterior MCA territory of the contralateral hemisphere. Therefore, a complete examination resulted in eight ROIs to be analyzed (Fig. 1a).

### Analysis of the CT Perfusion Measurements

From CT perfusion (Siemens Somatom Edge, Nürnberg, Germany), we measured the TTP and MTT of all segmented anatomic regions of 16 patients, namely the MCA and BG (OLEA Medical, France). We normalized the MCA against the BG regions, similar to UPI normalization (see “Normalization of UPI” section). All further analyses comparing CT TTP and MTT with UPI values were performed in concomitance with the analyses of UPI values. Detailed information is provided in the statistical methods section.

### Statistical Analysis

#### Normalization of UPI

The difference between left and right BG regions was performed by a linear regression model; significance was determined by Pearson’s product moment correlation coefficient. Further normalization of MCA regions (P_MCA_) was performed by relative scaling of the measurements in accordance with the BG perfusion (P_BG_).

$$P_{{{\text{MCA}}\;{\text{norm}}}} = \frac{{\frac{{\mathop \sum \nolimits_{i = 1}^{n} P_{{{\text{BG}}i}} }}{n}}}{{\frac{{\mathop \sum \nolimits_{i = 1}^{z} P_{{{\text{MCA}}i}} }}{z}}}$$

*P*, perfusion measurements; *n*, number of BG perfusion measurements; *z*, number of MCA perfusion measurements.

### Ratios of Baseline-Follow-Up Measurements

To calculate the difference between baseline perfusion (day 1–3) and later time points, we used the fold change (*P*_*T1,T2,…Tn*_*/P*_baseline_) to evaluate perfusion restriction. The Wilcoxon rank sum test was used to determine significance, and *p* value adjustment was performed by Benjamini-Hochberg.

### Tracking Therapeutic Intervention

Because UPI has not been validated as a tool for predicting cerebral tissue hypoperfusion and a gold standard for verifying tissue hypoperfusion has not been defined, we aimed to semiquantitatively assess tissue perfusion by comparing TTP values before and after spasmolysis (intraarterial injection of nimodipine).

### Prediction Model

To achieve a simple structuring of the information, we decided to implement a decision tree model with hierarchically structured information that passes through three filter layers to determine between high-risk and moderate-risk UPI examinations. We trained the decision tree using squared error as the cost function to estimate the split point within each layer. In the first layer, we evaluated the intraside difference between the anterior and posterior ROIs. The following layers two and three evaluate the perfusion ratios between the left and right MCA territory.

$${\text{Layer}}\,1: R_{{{\text{level}}1}} = \frac{{{\text{max}}\left[ {nP} \right]}}{{{\text{min}}\left[ {nP} \right]}}$$

$${\text{Layer}}\,2: R_{{{\text{level}}2}} = \frac{{{\text{max}}[{\text{mean}}(nP_{{{\text{left}}}} ),{\text{mean}}(nP_{{{\text{right}}}} )]}}{{{\text{min}}[{\text{mean}}(nP_{{{\text{left}}}} ),{\text{mean}}(nP_{{{\text{right}}}} )]}}$$

$${\text{Layer}}\,3: R_{{{\text{level}}3}} = \frac{{{\text{max}}\left[ {\min \left( {nP_{{{\text{left}}}} } \right),\min \left( {nP_{{{\text{right}}}} } \right)} \right]}}{{{\text{min}}\left[ {\min \left( {nP_{{{\text{left}}}} } \right),\min \left( {nP_{{{\text{right}}}} } \right)} \right]}}$$

*nP* defined the normalized TTP measurements of the defined ROIs.

In case a UPI examination showed ratios above at least one of the trained thresholds (layer 1 > factor 1.48, layer 2 > 2.1, layer 3 > 1.3), the examination was ranked as high-risk. In cases in which no ratios were found that defined patients as being high-risk, the examination was ranked as moderate-risk. The thresholds were tailored to high sensitivity of the model by accepting a high rate of false positive UPI measurements.

## Results

Between August 2013 and December 2015, we enrolled 30 patients into this prospective study. Twenty-four patients (80%) were women and six patients were men (20%). The median age was 54 years (interquartile range 48–67). In 28 patients (93%), the ruptured aneurysm was treated by endovascular coiling, and two patients underwent clipping. Twelve (40%) patients presented with a DIND. For further analyses of the UPI, we defined the minimum requirements of sequential examinations as follows: a baseline UPI examination (at day 1–3) and two follow-up UPI measurements at a median time point of 4–12 days and a late time point at 13–21 days. Of 30 patients, 27 met the inclusion criteria for sufficient follow-up (total of 130 UPI examinations), and the number of examinations per patient ranged from 3 to 9 examinations (Fig. 1a).

### Perfusion Midline Region Measurements Normalize Interpatient Variance

**Fig. 2:**
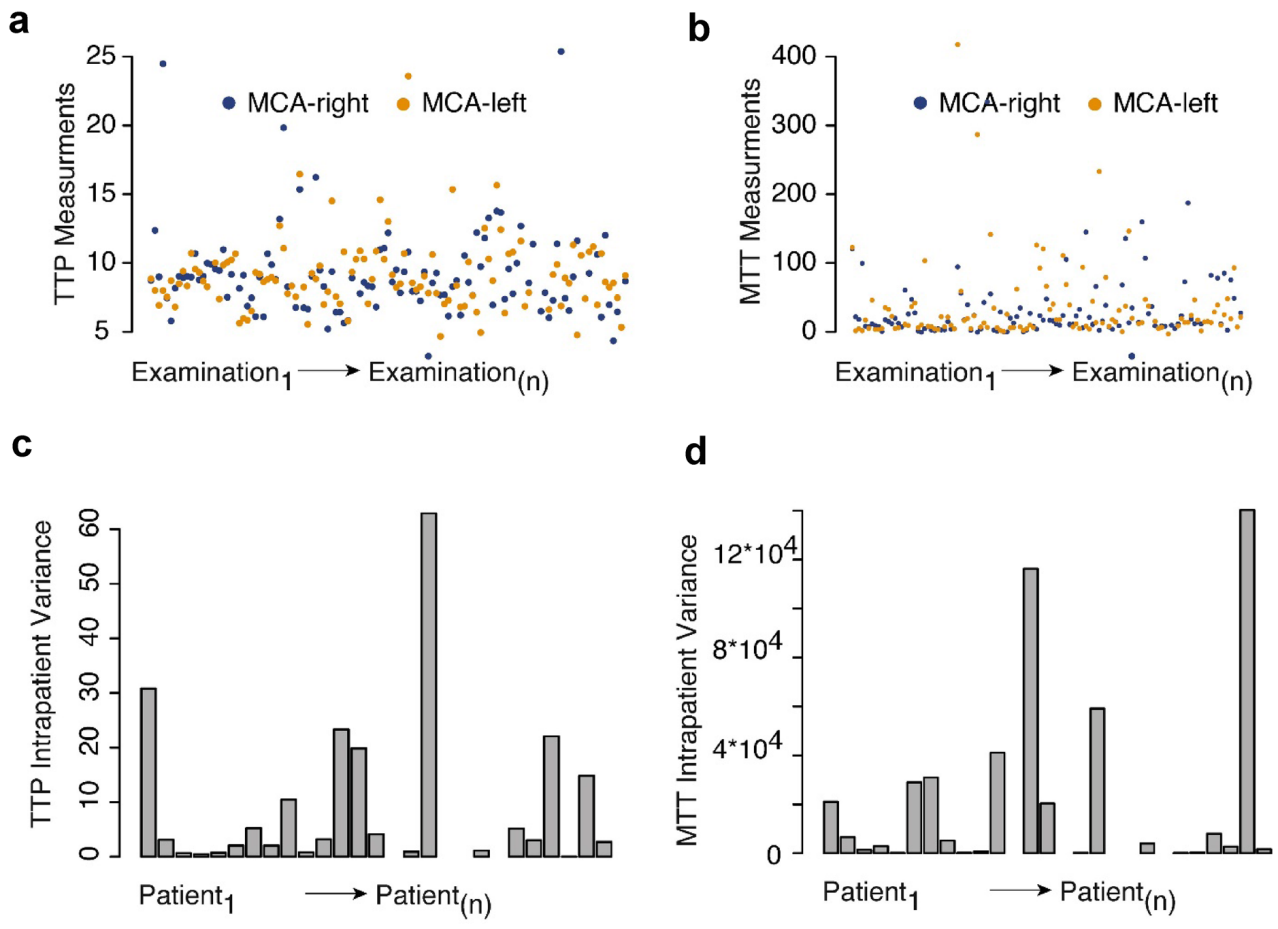
The interpatient variance of TTP (**a**) and MTT (**b**) values for the middle cerebral artery territories. In addition, intrapatient variance of TTP (**c**) and MTT (**d**) values are displayed. The variance of the MTT values is increased compared with TTP values. MCA, middle cerebral artery, MTT, mean transit time, TTP, time to peak

First, we benchmarked the robustness of the various UPI parameters across patients and measurements, which uncovered a strong interpatient and intrapatient variance of MTT and TTP values (Fig. 2a–d). To normalize multiple measurements, we used a relatively stable quotient of the right and left perfusion at the midline region ROI (Fig. 1b). Across all examinations, a strong correlation of the midline region ROI was found (*R*^2^ = 0.72, *p* < 0.001) for the TTP measurements but not for the MTT measurements (Fig. 1c–d). Based on the fact that MTT measurements revealed considerably more susceptible to disturbances during normalization and did not show robust measurements within multiple examinations we focused on TTP measurements in the further analysis. In the following sections, we refer to TTP as normalized TTP values.

### Tracking Therapeutic Intervention Prespasmolysis and Postspasmolysis

**Fig. 3:**
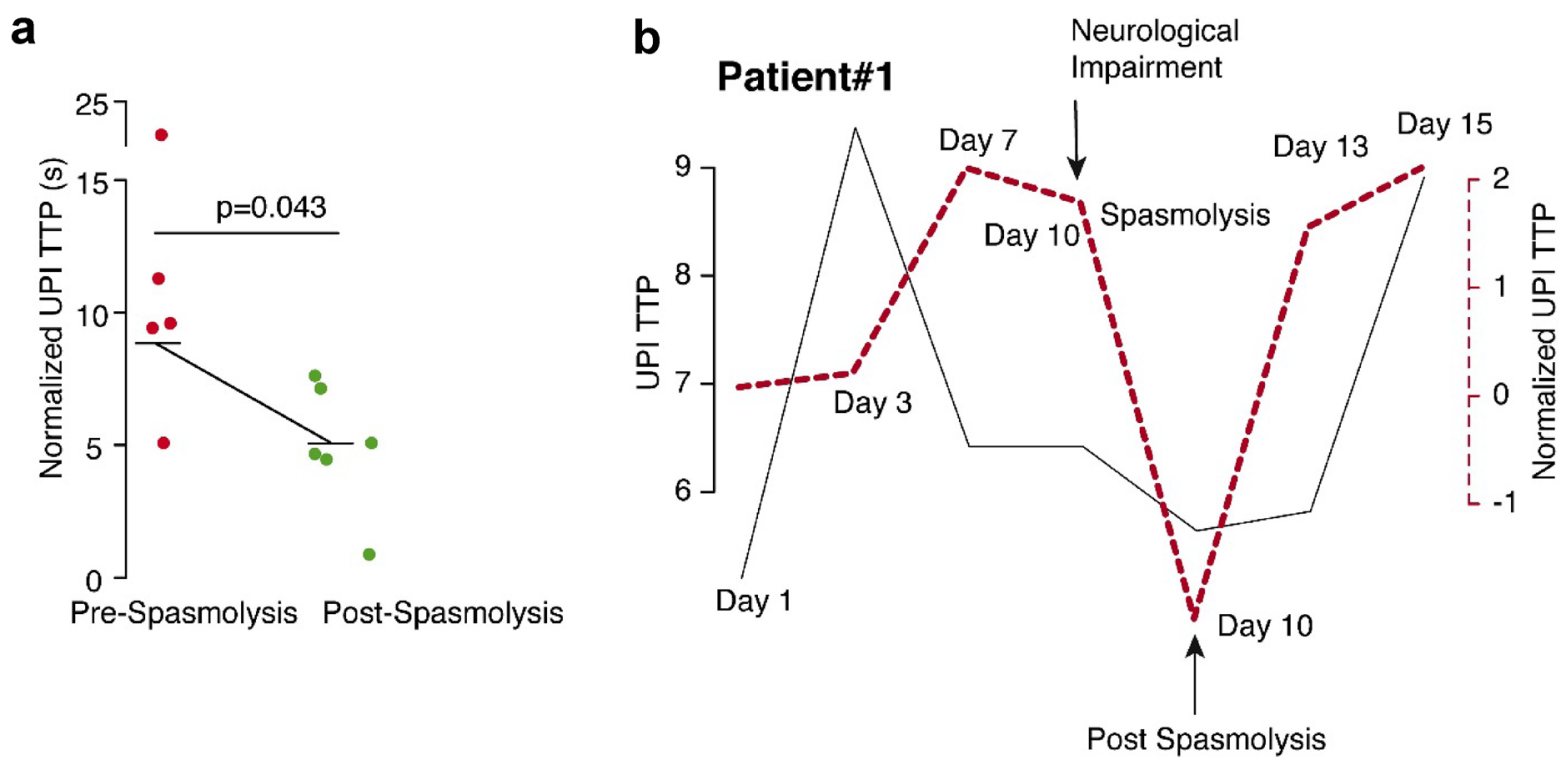
Analysis of TTP values in patients before (red) and after (green) spasmolysis (**a**). TTP values show a significant decline after spasmolysis. **b** Comparison between normalized (red) and nonnormalized (gray) TTP values in one patient (patient #1) before and after spasmolysis. Normalized values show a clear decrease in TTP values at day 10 after spasmolysis, which was indicated because of a new neurologic deficit. In contrast, nonnormalized values don’t reflect the effect of spasmolysis on TTP values. TTP, time to peak, UPI, ultrasound perfusion imaging

In Fig. 3, we present serial TTP measurements, including measurements before and after spasmolysis at day 10. In the cohort, four patients underwent a spasmolysis. All these patients showed a significant reduction of the TTP measurements (9.62-s delay vs. 6.07-s delay, *p* = 0.043) comparing prespasmolysis with postspasmolysis (Fig. 3a). Our data confirmed that normalized TTP values were more robust in tracking the effects of spasmolysis across multiple examinations (Fig. 3b).

### Normalization of MCA Territory and Longitudinal Observation

**Fig. 4:**
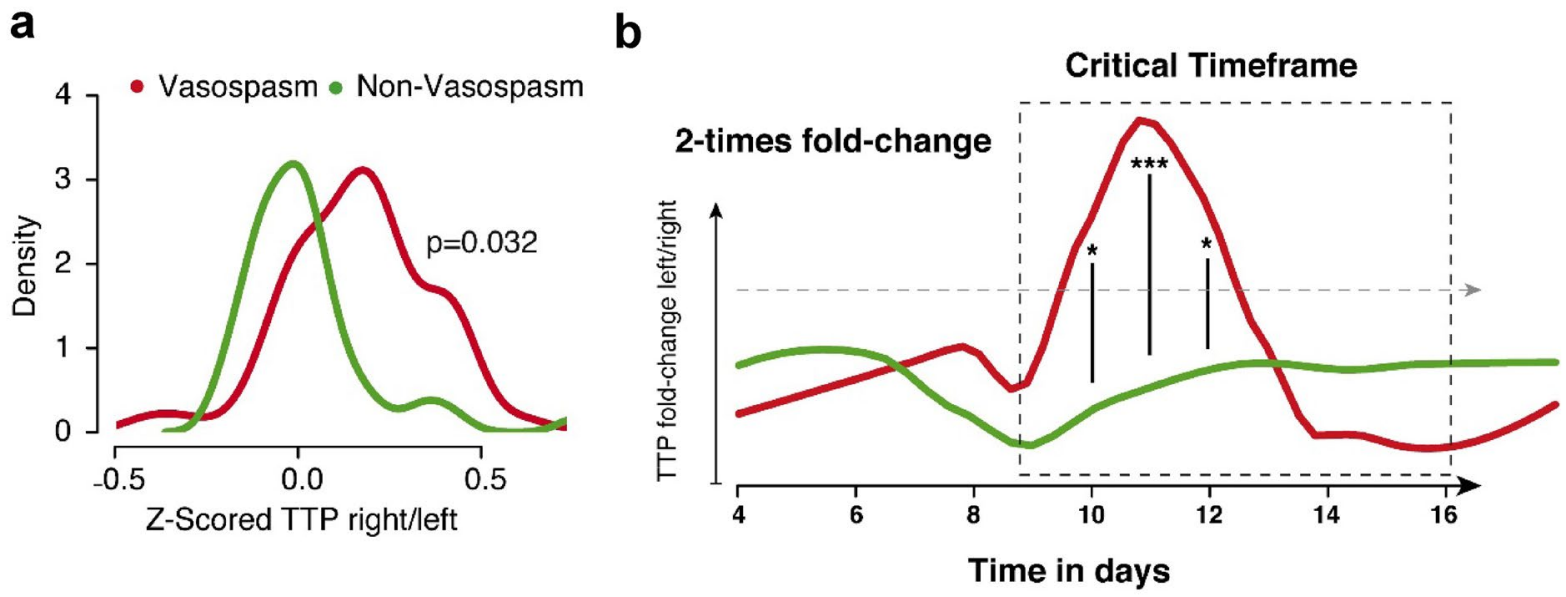
Comparison between left-to-right ratios of TTP values between patients who present a delayed ischemic neurologic deficit and those who do not (**a**). **b** Longitudinal analysis of TTP values between these two groups. Patients with a delayed ischemic neurologic deficit (red) show a significant increase of left-to-right ratios, especially between days 10 and 12. TTP, time to peak

**Fig. 5:**
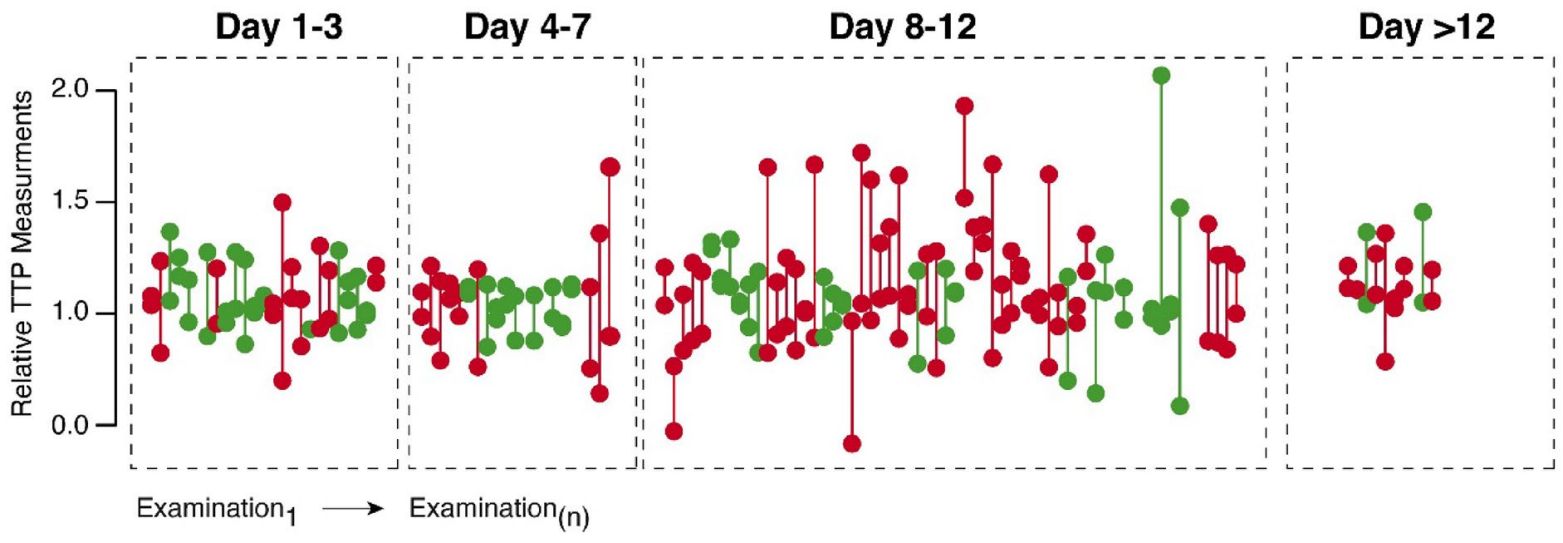
Illustration of left-to-right TTP ratios of all evaluated ultrasound perfusion examinations. Patients who present a delayed ischemic neurologic deficit are marked in red and those who presented without a delayed ischemic neurologic deficit are marked in green. Patients with delayed ischemic neurology deficit show an increase in TTP ratios, especially during the critical time period (8–12 days). TTP, time to peak

We assumed that vasospasm dominantly affect one MCA territory, which leads to interference within the regional examination areas of the left and right hemispheres. To investigate the extent to which perfusion measurements are influenced by this circumstance, we analyzed the ratios of left-to-right TTP values. In patients with DIND, we detected a significant change between left-to-right TTP ratios, particularly at the critical time frame between days 7–12. Patients with DIND showed a significant, 4.86 times increase of the left/right TTP ratio compared to a mean fold change in patients without DIND of 0.9 times (*p* = 0.032, Figs. 4a, 5).

### Comparison Between CT Perfusion and UPI

**Fig. 6:**
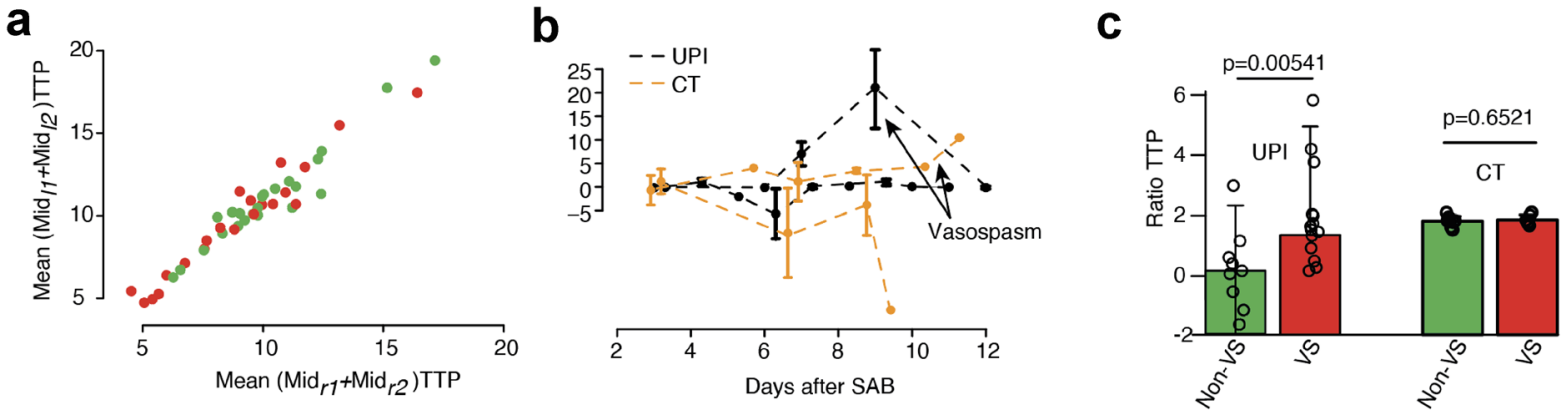
TTP CT imaging (left vs. right) measurements (red, with vasospasm; green, without vasospasm), correlation, and *p* values are determined by Pearson correlation coefficient (**a**). **b** Comparison between TTP longitudinal UPI (in black) and CT imaging (orange). **c** Barplot of differences between the left-to-right ratio within UPI (left) and CT (right) with vasospasm and without vasospasm. CT, computed tomography, TTP, time to peak, UPI, ultrasound perfusion imaging

In order to validate our data by the most broadly used imaging method to quantify cerebral perfusion, we compared CT perfusion at early (3 days) and late timepoints (8–12 days) with paired UPI measurements. Paired CT and UPI data were available for 16 patients with highly significant correlation between right and left midbrain regions (*R* 0.92, *p* < 0.001) confirming the accuracy of UPI-TTP measurements. From comparing the left-to-right ratio (*p* = 0.2) or longitudinal development of TTP changes, we did not observe significant differences in neither MTT nor TTP perfusion from CT imaging (Fig. 6).

### Results of UPI in Symptomatic Patients

On longitudinal measurements of the left–right difference (LR_Diff_) of TTP comparing patients with DIND (red) attributable to cerebral hypoperfusion with those without DIND (green), LR_Diff_ shows a significantly increase in patients with a DIND, with a peak at days 10–12 (*p* = 0.032) (see Fig. 4b).

Even though there is a pronounced variance of UPI measurements of the cohort, the parameter LR_Diff_ showed a robust differentiation between affected patients and nonaffected patients.

### Prediction Model for DIND Based on UPI Records

Although LR_Diff_ of TTP was found to predict tissue hypoperfusion, the overall accuracy was limited because of a pronounced heterogeneity within the cohort. Our data suggest that single parameters of UPI may allow a prediction of tissue hypoperfusion leading to DIND. To improve the accuracy of our UPI-based prediction of DIND, a multilayer prediction model was designed in which each UPI examination was evaluated at multiple levels and rated based on the estimated risk of DIND. We decided to evaluate single UPI examinations and not stratify patients in our prediction model because patients with DIND revealed nonpathological perfusion at baseline examinations.

**Fig. 7:**
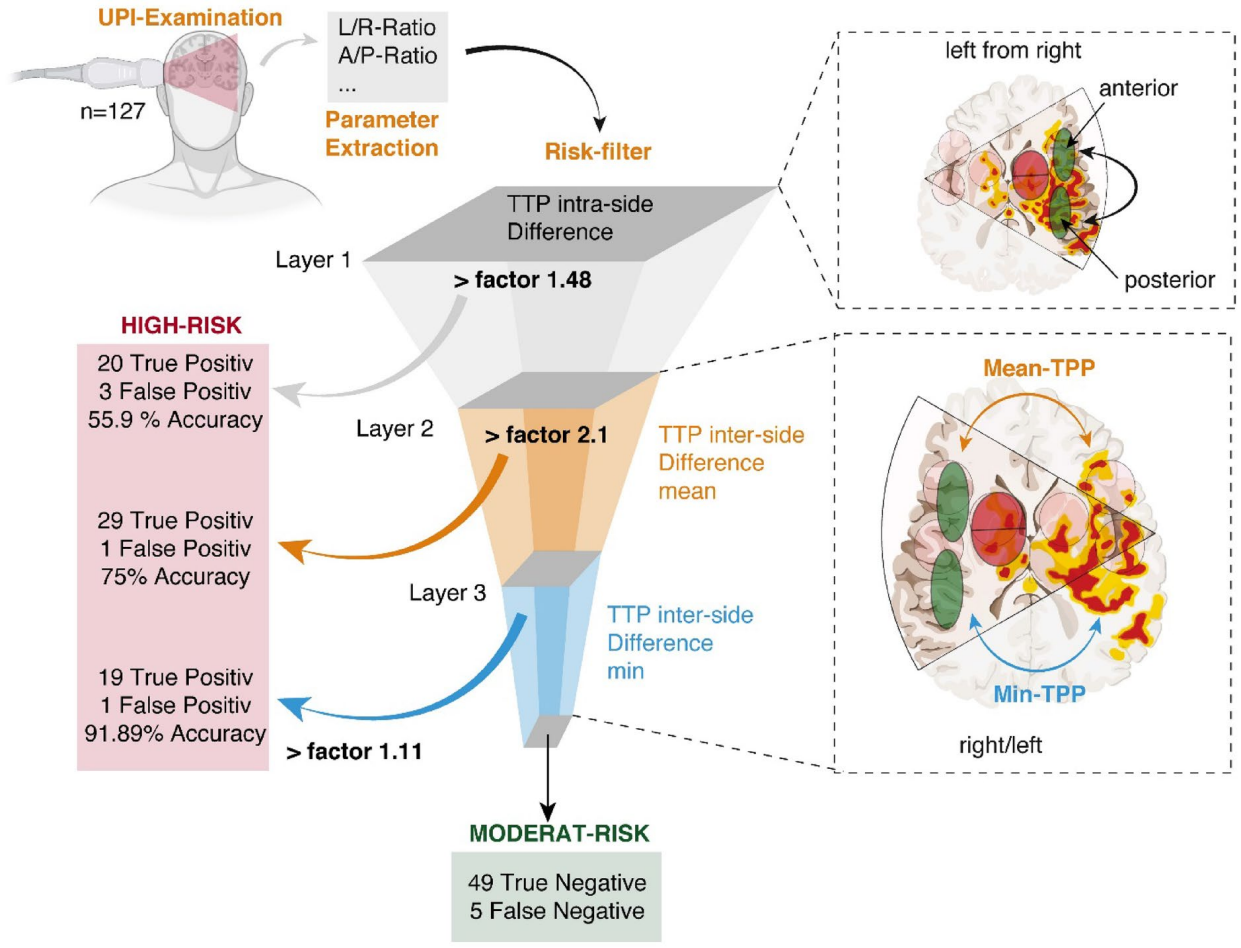
Decision tree model to sort out patients at risk for DIND. One layer must be positive to be graded as high-risk. A/P-Ratio, anterior/posterior ratio, L/R-Ratio, left-to-right ratio, Min, minimum, TTP, time to peak

**Fig. 8:**
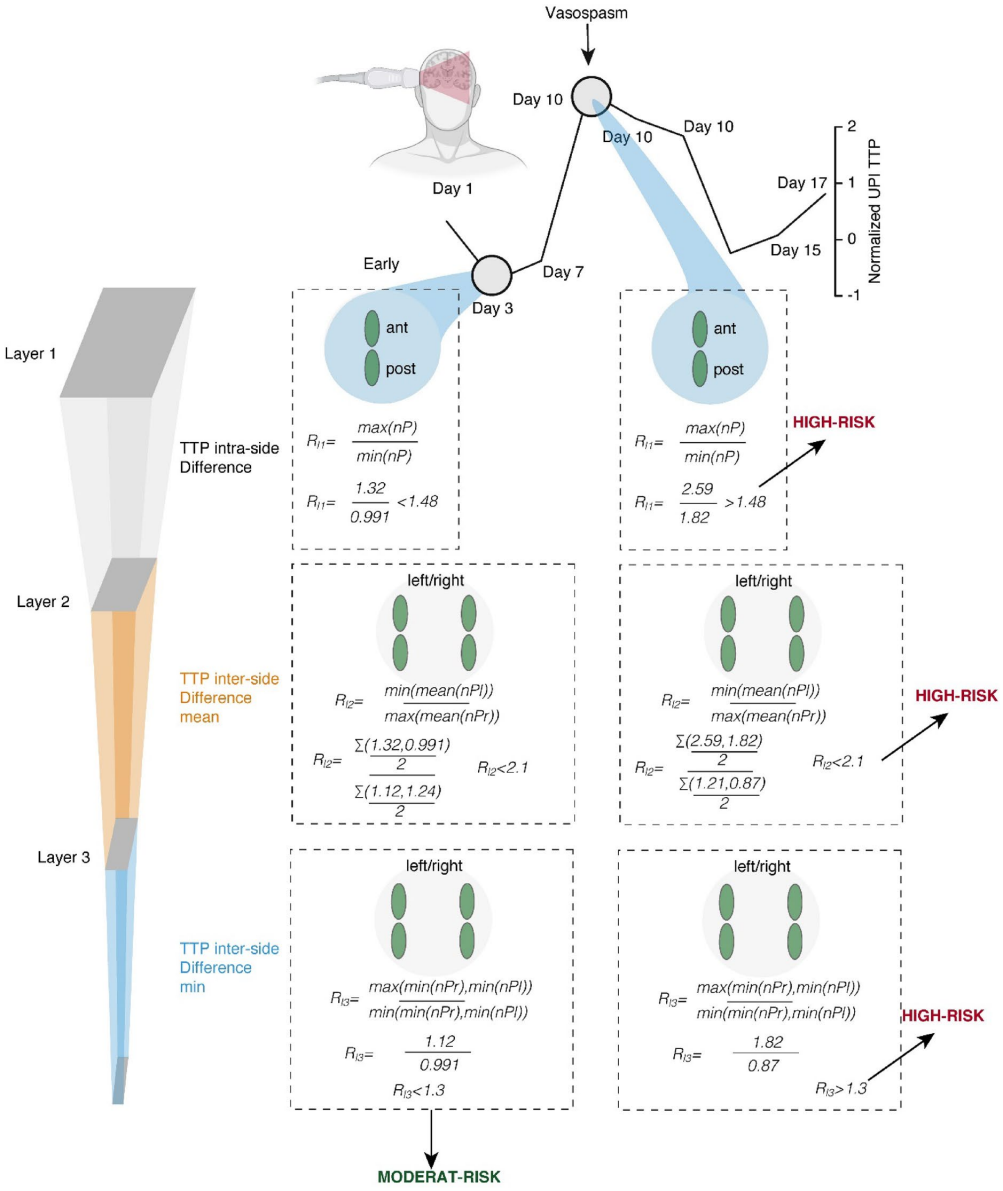
Example of two patients being graded according to our decision tree model. min, xxx, max, xxx, nP, xxx, TTP, time to peak, UPI, ultrasound perfusion imaging

We established a decision tree model to select patients at risk for DIND (Fig. 7, Table 1). To be classified as high-risk, a UPI examination must have met at least one of the following criteria. At the first layer, we estimated the difference between anterior and posterior TTP measurements (intraside TTP difference). UPI examinations (*n* = 127) with a ratio between anterior and posterior TTP values more than 1.48 were found to be at risk for DIND, with an accuracy of 55.9% (sensitivity 37.7% and specificity 94.4%). In the second layer UPI examinations that passed the first layer (*n* = 104) were evaluated based on the above explained left-to-right TTP ratio (interside difference). Ratios of the difference in mean higher than 2.1 were found to be highly associated with DIND (accuracy 75%, sensitivity 56%, and specificity 98%). In the third layer, examinations that passed the first two layers (*n* = 74) were evaluated based on the left-to-right difference of the minimal TTP values within all examined regions. If the ratio between left and right was higher than 1.11, the UPI examination was ranked as high-risk (accuracy 91.89%, sensitivity 79%, and specificity 98%). The model was tailored to minimize the number of false negative evaluations. In our cohort, the global accuracy of our decision model was 92%, with a sensitivity of 93.15% and a specificity of 90.7% (Fisher’s exact test, *p* < 2.2 × 10^−16^. Examples of patients are given in Fig. 8.

**Table 1:** Contingency table for every layer of the decision treee model

Layer	Positive examinations, *n*	Negative examinations, *n*	TotalUPI, *n*	Parameter (%)
Layer 1:	–	–	127 UPI	Accuracy,: 55.9%
Predicted positive	20	3	23	Specificity,: 94.4%
Predicted negative	53	51	104	Sensitivity,: 37.7%
Layer 2:	–	–	104 UPI	Accuracy,: 75%
Predicted positive	29	1	30	Specificity,: 98%
Predicted negative	24	50	74	Sensitivity,: 56%
Layer 3:	–	–	74 UPI	Accuracy,: 91.89%
Predicted positive	19	1	30	Specificity,: 98%
Predicted negative	5	49	54	Sensitivity,: 79%
Total model	–	–	127 UPI	Accuracy,: 92%
Predicted positive	68	5	73	Specificity,: 90.7%
Predicted negative	5	49	54	Sensitivity,: 93.15%

*UPI* ultrasound perfusion imaging

## Discussion

This is the first prospective study evaluating the capability of UPI to detect cerebral hypoperfusion in the setting of aSAH. The results of this first study are threefold. First, we could show that there is a strong correlation of the midline ROIs for the TTP examination and that these measurements can be used to normalize the ROIs of the MCA territory. This normalization is necessary because of the high variance of raw signals and leads to a reduction of unspecific bias of individual measurements. In aSAH perfusion restrictions of the midline regions are conceivable and insufficiently investigated. Up to date most studies have been performed in patients with ischemic stroke [15–17]. Secondly, the data show that the LR_Diff_ of the minimal obtained perfusion does most likely predict a cerebral hypoperfusion. Lastly, we could present a three-layer prediction model for cerebral hypoperfusion based on these first results.

In assessable patients, the clinical examination is the mainstay of diagnosing DIND. Yet, patients with aSAH are often severely impaired or even sedated, intubated, and clinical examination is of limited use. Additional examinations are used to diagnose possible vasospasms. Transcranial doppler sonography (TCD) is the mainstay in bedside detection of cerebral vasospasm [11, 12]. Despite its wide use, it has important drawbacks. There is a lack of evidence of the usefulness of TCD and only low or very high mean MCA flow velocities (i.e., < 120 or ≥ 200 cm/s) reliably predicted the absence or presence of clinically significant angiographic vasospasm [18, 19]. In addition, assessment of TCD is observer-dependent, requires regular practice, and depends on an adequate acoustic bone window [20, 21]. TCD only measures the surrogate parameter, flow velocity, and no tissue perfusion. Compared with UPI assessments, TCD and UPI share some drawbacks. In this investigation, we have only assessed the MCA territory, and a UPI examination also requires a learning curve and is, to some part, observer-dependent. UPI measurements has some advantage in patients with inadequate temporal bone window, because the application of i.v. contrast enables examinations when conventional TCD examinations fail. Yet, the main advantage is the evaluation of tissue perfusion rather than a surrogate parameter. Other methods such as CT perfusion (CTP) or magnetic resonance tomography perfusion can assess relative tissue perfusion [22] but pose additional risks to the patient. Mainly transport of the intensive care patient, flat body position in patients with critical intracranial pressure elevation, x-ray exposure, intolerances and additional high costs. UPI can avoid some of these risks because the examination can be performed in the intensive care unit and on patients in different body positions. It does not require x-ray or additional resources, such as scanning time, and is therefore in any order repeatable. For long, arterial narrowing on digital subtraction angiography was the accepted gold standard for arterial vasospasm. Despite a correlation between arterial diameter and proximal cerebral circulation time (distal internal carotid artery and M4 segment of the MCA), this could also not be proven for arterial diameter and parenchymal cerebral circulation time [23]. In addition, arterial narrowing on digital subtraction angiography (DSA) does not always correlate with DIND or DCI [24, 25]. Therefore DSA, seems to display proximal arterial narrowing yet adds limited value to the diagnosis of peripheral vasospasm and actual tissue perfusion.

The aforementioned techniques to detect cerebral vasospasm and hypoperfusion in the setting of SAH have been evaluated in various studies including systematic reviews. Compared to our decision model (sensitivity 93.15%, specificity 90.7%) the sensitivities and specificities are reported to be 90% and 71% for TCD [26], 79.6% and 93.1% for CTA [27], and 74.1% and 93% for CTP [27]. Data on sensitivity and specificity for perfusion weighted magnetic resonance imaging are not available. In addition, many studies indicate DSA as being the gold standard for detection of cerebral vasospasm, therefore yielding no data either. The limitation of the current literature is that with respect to TCD the single studies have defined different cutoff values for cerebral vasospasm [26], for CTP studies different perfusion parameters and different thresholds are reported to have the highest diagnostic yield [28, 29] and in general the definition of cerebral vasospasm is far from uniform [26].

Our proposed decision tree should be regarded as a first approach to sort out patients who are at risk. Because of the limited amount of included UPI measurements this decision tree should not be regarded as final.

An optimal screening tool for patients with aSAH to prevent DCI would be capable to assess tissue perfusion, be performed bedside, be repeatable, without additional x-ray exposure, and low costs. In this first prospective study, we could show that UPI meets these requirements. Despite this first encouraging results this methods needs further investigation in larger prospective trials.

The limitations of this study are its single center design and the limited number of included patients. The study team performing the UPI examinations was not blinded for clinical course. In addition, DIND is a subjective outcome measure and was only present in 12 out of 27 patients, which might introduce a bias.

## Conclusions

In conclusion, the assessment of UPI to enable detection of cerebral hypoperfusion after aSAH is feasible. What is most indicative of this is the left–right difference of TTP values. We propose a multilayer model to define patients at a high risk for cerebral hypoperfusion. Further prospective studies are required prior to routine clinical use of UPI measurements to detect cerebral hypoperfusion in patients with SAH.

## Clinical Trial registration

ClinicalTrials.gov: NCT02907879.
